# Low Power Optoelectronic Neuromorphic Memristor for In‐Sensor Computing and Multilevel Hardware Security Communications

**DOI:** 10.1002/advs.202524299

**Published:** 2026-03-09

**Authors:** Bo Sun, Jinhao Zhang, Jialin Meng, Tianyu Wang

**Affiliations:** ^1^ Shandong Key Laboratory of Next‐Generation Semiconductor Technology and Systems School of Integrated Circuits Shandong University Jinan China; ^2^ Shenzhen Research Institute of Shandong University Shenzhen China; ^3^ Suzhou Research Institute of Shandong University Suzhou China; ^4^ National Integrated Circuit Innovation Center Shanghai China; ^5^ State Key Laboratory of Crystal Materials Shandong University Jinan China

**Keywords:** hardware security communication, in‐sensor computing, memristor, neuromorphic computing, optoelectronic device

## Abstract

Conventional software‐based encryption faces mounting limitations in power efficiency and security, inspiring the development of emerging neuromorphic computing hardware encryption. This study presents a hardware‐level multi‐dimensional encryption paradigm utilizing optoelectronic neuromorphic devices with low energy consumption of 3.3 fJ, exhibiting great potential in motion detection, in‐sensor computing and multilevel encrypted information communication. By encoding ASCII characters into unique optical pulse sequences defined by wavelength, duration, and pulse number, the device transforms digital information into physically obfuscated electrical responses, thereby establishing a secure encryption mechanism. Based on neuromorphic response of optoelectronic device, convolutional neural network was trained to decrypt signals with recognition accuracy of 97.4% for legitimate users while maintaining robustness against unauthorized access (∼2.88% accuracy). To address complex real‐world scenarios of maritime communication, dual‐authentication “friend‐or‐foe” identification system was constructed with two‐layer authentication. The neuromorphic optoelectronic system combines motion perception, real‐time flag semaphore recognition via reservoir computing with multi‐band photonic encryption, showing great potential in next‐generation neuromorphic maritime communication.

## Graphical abstract

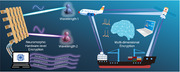

## 1 Introduction

In the era of artificial intelligence (AI) and big data, the enormous computational power required by AI poses significant challenges to the conventional von Neumann architecture [1, 2, 3]. The separation of memory and processing in this architecture leads to substantial energy waste during data transfer, a problem that becomes particularly pronounced as data volumes continue to grow [4]. This has resulted in the so‐called “power wall” and “memory wall” challenges [5]. To overcome these limitations, a new computing paradigm—computing‐in‐memory (CIM)—has been proposed based on non‐volatile memristors [6, 7, 8]. Memristors have been demonstrated to emulate neurons or synapses, enabling neuromorphic computing [9, 10]. Moving beyond computing‐in‐memory, in‐sensor computing processes data directly where it is captured [11, 12, 13]. This reduces the energy and latency costs of conventional sense–transmit–process systems. By embedding memory and computation into the sensor, analog signals can be preprocessed before digitization, lowering data transfer and enabling efficient perception [14]. Optoelectronic memristors, which convert light into programmable synaptic responses, are promising for such systems. However, current work focuses mainly on single tasks like image preprocessing, while integrated systems that combine multi‐wavelength sensing, in‐sensor computing, and hardware security remain largely unexplored.

Meanwhile, information security has become an issue of increasing concern. Conventional secure communication and encryption typically rely on software algorithms that use complex mathematical methods to render data unreadable by unauthorized parties [15]. However, with the rapid advancement of AI, purely mathematics‐based encryption appears increasingly vulnerable. Moreover, the high computational cost associated with complex encryption algorithms also limits their scalability [16]. In response, researchers have begun shifting encryption principles from purely mathematical approaches to interdisciplinary combinations of mathematics and physics. As a result, hardware encryption has attracted growing attention. A number of studies on hardware encryption have been reported, including several that leverage memristive devices [17, 18, 19, 20]. Most of these approaches, however, rely on the randomness and instability of conductive filament (CF) formation and rupture within memristors [21]. Such stochasticity is harnessed as a source of entropy for physically unclonable functions (PUFs) [22] or true random number generators (TRNGs) [23]. These are innovative concepts that achieve high security with low power consumption. Nevertheless, such schemes are susceptible to environmental variations (e.g., temperature, humidity) that cause fluctuations in entropy, and are also affected by non‐ideal noise in circuits, which may necessitate additional complex filtering circuitry—increasing both manufacturing difficulty and power consumption [24]. Therefore, developing new hardware encryption strategies based on the intrinsic properties of memristors remains a worthwhile research direction.

Optoelectronic synaptic devices, which can be modulated by both optical and electrical signals, are capable of emulating information transmission and synaptic plasticity between neurons [25, 26]. They thus provide an efficient and low‐power hardware foundation for neuromorphic computing [27, 28]. Optoelectronic memristive devices have been widely studied due to their simple structure. As two‐terminal synaptic devices, their conductance changes nonlinearly with external stimuli and can be retained within the device [8, 29, 30, 31, 32, 33, 34]. Hence, they are often investigated primarily as neuromorphic devices, with relatively less attention paid to other potential applications. Generally, optoelectronic synaptic devices can respond differently to light across multiple wavelengths, and dynamic optical pulses can carry information [35, 36, 37, 38, 39]. This principle enables an encryption scheme that encodes data in different light bands and controls optoelectronic synaptic devices with optical pulses for secure communication.

In this work, we constructed an optoelectronic synaptic array based on a PET/ITO/HfAlO_x_/NbO_x_/ITO material system. Using this array, we designed a hardware encryption method—multi‐dimensional encryption—and successfully integrated decryption functionality into a microcontroller for portable information processing. The devices exhibited a range of neurosynaptic behaviors under optical pulse modulation, including excitatory post‐synaptic current (EPSC), spike‐number‐dependent plasticity (SNDP), spike‐rate‐dependent plasticity (SRDP), spike‐width‐dependent plasticity (SWDP), and the transition from short‐term plasticity (STP) to long‐term plasticity (LTP). It is also worth highlighting that the devices not only responded to optical stimuli across multiple wavelengths but also demonstrated excellent power efficiency, with ultralow energy consumption per switching event. Inspired by these characteristics, we proposed an information encryption method that leverages the device's sensitivity to optical pulse parameters—such as wavelength, duration, and number of pulses—to enhance data transmission security in IoT applications. In this encryption scheme, these parameters serve as carriers of information input into the array. The optoelectronic memristor array maps the inputs into unique response signals based on its physical properties, thereby encrypting the information. We then employed a convolutional neural network (CNN) to decrypt the information, completing an end‐to‐end pipeline covering conversion, transmission, encryption, and decryption. Furthermore, we deployed the trained CNN onto a microcontroller, demonstrating the feasibility of secure communication on mobile devices.

## 2 Result and Discussion

**FIGURE 1:**
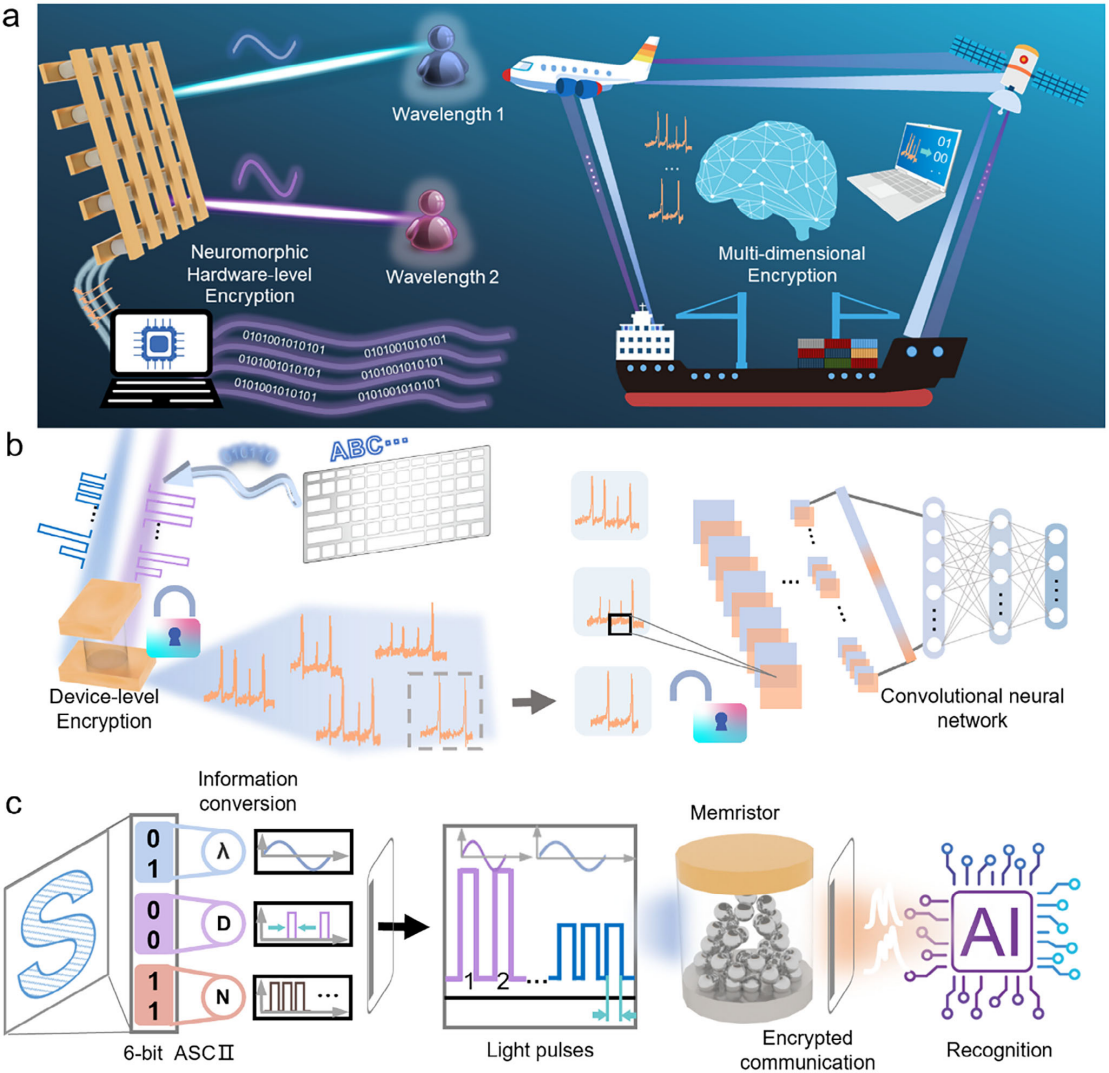
Schematic diagram of device‐level dual‐band encryption. (a) Illustration of the principle and application of device‐level dual‐band encryption (The right panel of Figure 1a shows a schematic of a potential application scenario). (b) Overview of the entire device‐level dual‐band encryption process, including the encryption process (left) and the decryption process (right). (c) Detailed schematic of the device‐level dual‐band encryption process, covering information conversion, transmission, encryption, and decryption.

Figure 1 illustrates the encryption method and process designed based on our neuromorphic device. In brief, encryption relies on the device's distinct responses to different incident optical pulses. A unique combination of optical pulses results in a uniquely determined electrical response from the device. Thus, using this neuromorphic device as a platform, we propose a novel hardware encryption method.

As shown in Figure 1a, when light of two wavelengths illuminates the device array in a specific sequence, the device produces a unique electrical response. By recognizing this electrical signal using a convolutional neural network (CNN), the original encrypted information can be recovered, thereby achieving decryption. The right side of Figure 1a demonstrates potential applications of this method, such as enabling secure multi‐terminal communication across marine, aerial, and space domains. Figure 1b presents the complete encryption and decryption workflow. The left side depicts the encryption process, which consists of three stages: information conversion, transmission, and device reception. The information to be transmitted is first converted into binary form, then transformed into a sequence of optical pulses. This pulse sequence is applied to the device, and the resulting electrical response is collected, completing the encryption process. The decryption process, shown on the right side of Figure 1b, involves feeding the collected electrical signals into a pre‐trained CNN to decode the information.

Figure 1c provides a detailed schematic of the encryption process, using the letter “S” as an example. The corresponding 6‐bit ASCII code for “S” is “010011”, which is divided into three segments: “01”, “00”, and “11”. The first two bits represent the wavelength (λ) of the optical pulse—here, “01” corresponds to 450 nm. The middle two bits indicate the pulse duration (D)—“00” corresponds to 100 ms. The final two bits specify the number of pulses (N)—“11” corresponds to 30 pulses. Each unique binary sequence determines a unique set of optical pulse parameters, completing the conversion from alphanumeric information to optical pulse information. This optical pulse sequence, carrying the transmitted information, is then input into the device to obtain a uniquely corresponding electrical response, thereby finalizing the encryption. The encrypted electrical signal is subsequently decrypted by processing its image through a pre‐trained CNN, accomplishing the full procedure of encrypted communication.

**FIGURE 2:**
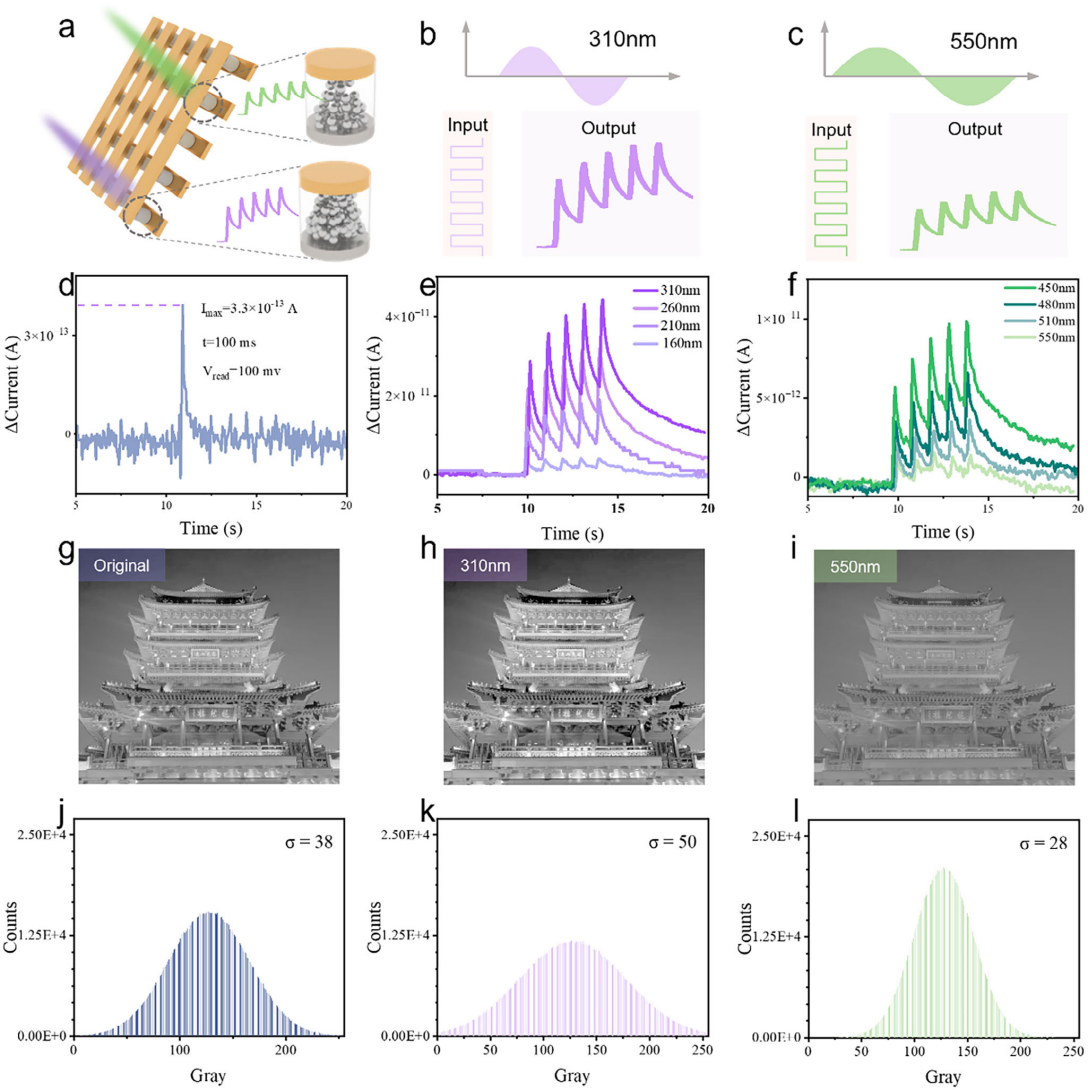
(a) Electrical response characteristics of the neuromorphic device under optical pulse stimuli of different wavelengths. (b,c) correspond to optical pulse stimuli at wavelengths of 310 and 550 nm, respectively. (d) Optimal power consumption performance of the device. (e) The minimum wavelength limit to which the device can respond in the short‐wavelength range. (f) The maximum wavelength limit to which the device can respond in the long‐wavelength range. (g) Grayscale image of the Jinan Chaoran Tower. (h) and (i) Processed results of photocurrent responses based on optical pulses at 310 and 550 nm wavelengths, respectively. (j–l) Grayscale value distribution profiles of the three images.

In this study, we fabricated a neuromorphic device with a PET/ITO/HfAlO_x_/NbO_x_/ITO structure on a PET/ITO substrate using conventional front‐end integrated circuit processes. The device structure is shown in Figure 2a. The fabrication process is illustrated in Figure S1, and XPS analysis results are presented in Figure S2. We conducted relevant tests for neuromorphic computing on the device. Figures S3a and S4 demonstrate the PPF characteristics of the device, indicating its ability to emulate biological plasticity. Additionally, Figure S3b–d exhibit the spike‐number‐dependent plasticity (SNDP), spike‐duration‐dependent plasticity (SDDP), and spike‐rate‐dependent plasticity (SRDP) of the device, respectively, demonstrating its capability to mimic biological learning and memory functions, thereby achieving a transition from short‐term plasticity (STP) to long‐term plasticity (LTP), as shown in Figure S3e. Furthermore, test results under different parameters are provided in Figures S4–S7. The transition from STP to LTP simulates the key process of consolidation from “short‐term memory” to “long‐term memory” in biological synapses: when the presynaptic terminal receives sparse stimuli, brief calcium influx only causes transient neurotransmitter release, corresponding to carriers in the device being rapidly captured by traps or undergoing recombination, leading to a quick decay of current to the initial state (STP). Under high‐intensity or high‐frequency stimulation, sustained calcium influx triggers further cellular changes, inducing physical remodeling and strengthening of the synaptic structure, which corresponds to a significant increase in the relaxation time of the device due to deep carrier accumulation or ion migration (LTP). This mechanism not only endows the device with brain‐like learning capability but also makes the encryption process analogous to biological memory formation—the key is encoded in specific stimulus patterns (combinations of pulse parameters), while the ciphertext corresponds to the unique and irreversible physical fingerprint formed within each device. Thus, security is tightly integrated with the inherent particle dynamics and physical uniqueness of the device. Inspired by these findings, the EPSC modulated by optical spiking pulses can also be applied to simple encryption communication tasks. Specifically, 100 ms optical spikes and no stimulation represent binary “1” and “0”, respectively. Based on ASCII code mapping, 26 English letters were successfully generated, with results displayed in Figures S8–S10.

To enable more complex information encryption using the device, it is first necessary to ensure that the device can respond to optical pulses of multiple wavelengths. Therefore, investigating its responsiveness across various wavelength bands is crucial.

The magnified detail on the right side of Figure 2a illustrates the differences within the device after stimulation with optical spikes at wavelengths of 550 and 310 nm, respectively. The input optical pulse information and the output electrical signals are displayed in Figure 2b,c. As shown, we used five consecutive optical pulses as input signals to verify the device's responsiveness to multiple wavelength bands. Each pulse had a width of 100 ms, with an interval of 900 ms between consecutive pulses. Additionally, during this process, we conducted a series of tests using single optical spike pulses to determine the device's optimal energy consumption performance (Figure 2d). Test results for other pulse durations are shown in Figure S11A–I. The tests revealed that the optimal energy consumption per synaptic event of the device is only 3.3 fJ, which is even lower than the 10 fJ per synaptic event of the human nervous system. It is important to note that this result was obtained under idealized test conditions and does not imply that the overall energy consumption of the device surpasses that of the human brain. However, it sufficiently demonstrates the significant potential of this synaptic device for low‐power neuromorphic computing.

Figure 2e,f shows the device's limiting response wavelengths in the short‐wavelength and long‐wavelength ranges, respectively. The device exhibits certain responsiveness to both 160 nm ultraviolet light and 550 nm green light, which serves as a physical basis for its applicability in multi‐dimensional encryption.

Furthermore, we observed that optical stimuli of different wavelengths can produce current waveforms with significantly varying amplitudes. Leveraging these differences in current amplification, preprocessing operations such as image contrast enhancement can be achieved. We conducted a proof‐of‐concept experiment using a grayscale image of the Jinan Chaoran Tower (Figure 2g). The original grayscale distribution of the image, shown in Figure 2j, follows a normal distribution with a standard deviation (σ) of 38. Under 310 nm optical spike stimulation, the device's current rises rapidly, with each pulse inducing a current value significantly higher than the previous one, demonstrating a contrast‐enhancing effect. As shown in Figure 2h, compared to Figure 2g, the brightness and darkness contrasts are more distinct. The grayscale distribution (Figure 2k) still follows a normal distribution, but with a larger standard deviation (σ = 50). The broader distribution of grayscale values, ranging from bright to dark, results in an image with higher contrast. In contrast, under 550 nm wavelength stimulation, the current increases more gradually. Figure 2i shows the image processing results based on the 550 nm wavelength sensitivity, with its grayscale distribution shown in Figure 2l. The smaller standard deviation (σ = 28) indicates that the grayscale values are more concentrated around the mean, resulting in an image with lower contrast and fewer noticeable noise points. By simulating spatial domain image processing that alters pixel grayscale distributions based on incident wavelength sensitivity, we demonstrate the image preprocessing functionality inspired by the retina.

Based on the above discussion, it can be concluded that the device exhibits distinct responses and behaviors to optical spike stimuli with different wavelengths, numbers, pulse widths, and frequencies. This variability establishes the physical foundation for device‐level encryption, as different optical pulse sequences can be uniquely mapped to specific electrical signal sequences. Accordingly, we have designed a novel neuromorphic hardware encryption scheme.

**FIGURE 3:**
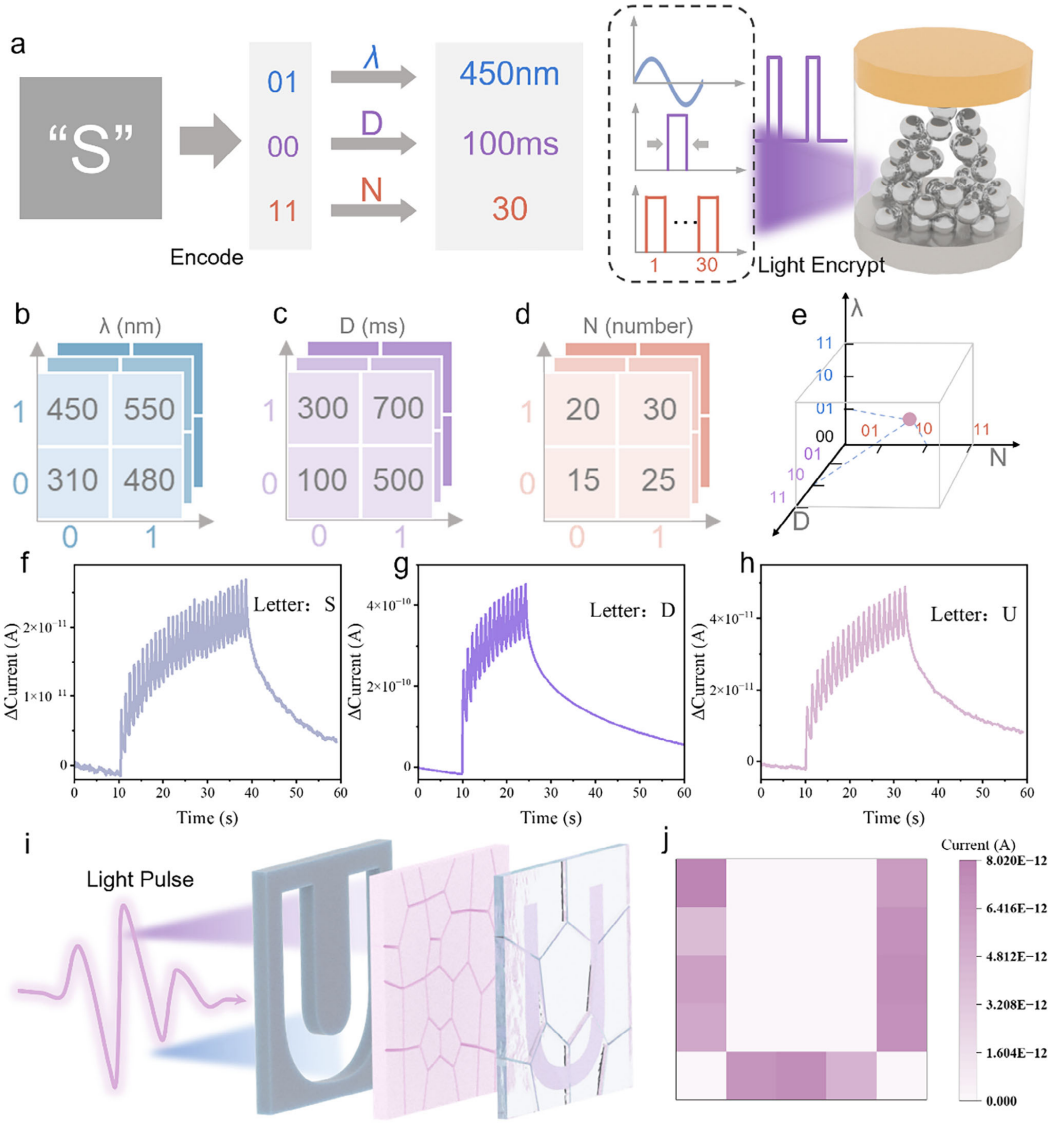
(a) The specific encryption process of multi‐dimensional encryption using the letter “S” as an example. (b,c) Correspondence tables illustrating the physical meanings mapped by the first, middle, and last two digits of the 6‐bit ASCII code. (e) Schematic diagram of the physical space corresponding to the integration of 3D information. (f–h) Electrical signal waveforms obtained based on the above mapping rules. (i) The letter “U” directly written into the device array using a mask. (j) Current distribution of the device array corresponding to the letter “U”.

As illustrated in Figure 3a using the letter “S” as an example, the encoding rules and encryption methodology of this hardware‐based approach are demonstrated. First, in the 6‐bit ASCII code table, the binary representation of “S” is “01‐00‐01”. This 6‐bit binary sequence is divided into three segments: the first two bits, the middle two bits, and the last two bits, which are mapped to a wavelength of 450 nm (λ = 450 nm), a single pulse duration of 100 ms (D = 100 ms), and a total of 30 pulses (N = 30), respectively. When this specific set of optical pulses is applied to the device, a corresponding electrical signal is generated. This electrical signal uniquely represents the letter “S” and can be used for encrypted communication. In this encryption process, information is first encoded into a binary sequence based on specific rules. By splitting this binary sequence and establishing relationships between the segments and physical parameters, the information is effectively transformed. The converted optical pulse information is then applied to the device, and the resulting current values are collected to obtain an electrical signal sequence, thereby completing the multi‐dimensional encryption of a single letter.

The correspondence tables in Figure 3b–d explicitly define these mappings for wavelength, duration, and pulse number, respectively. Crucially, this design intrinsically expands the encoding dimensions compared to conventional binary transmission. Even with only 6 bits of digital input, the combination of three independent physical parameters creates a richer stimulus space that is far more difficult to exhaustively probe or replicate than a simple digital key. Figure 3e synthesizes these individual dimensions into a unified 3D parameter space, visually emphasizing that every character occupies a distinct coordinate (λ, D, N) within this space. A flattened version of the full lookup table is provided in Figure S12. When this specific optical pulse set is applied to the neuromorphic device, it elicits a characteristic, non‐linear electrical response that serves as the physical ciphertext. This completes the encryption process: information is converted from digital bits to optical parameters, and then to a device‐specific electrical signature.

Following the above rules, we tested all 26 English letters and conducted multiple tests to facilitate subsequent neural network training. The test results for the letters “S”, “D”, and “U” are shown in Figure 3f,g, while the results for the remaining letters are provided in Figures S13–S15. Additionally, based on this mapping rule and using shadow masks, patterned illumination was applied to the device array. As demonstrated in Figure 3i, the letter “U” was successfully transferred onto the device array. The current distribution across the array, depicted in Figure 3j, clearly reveals the letter “U” through measured currents in each device. The current distributions for the letters “S” and “D” are shown in Figure S16.

**FIGURE 4:**
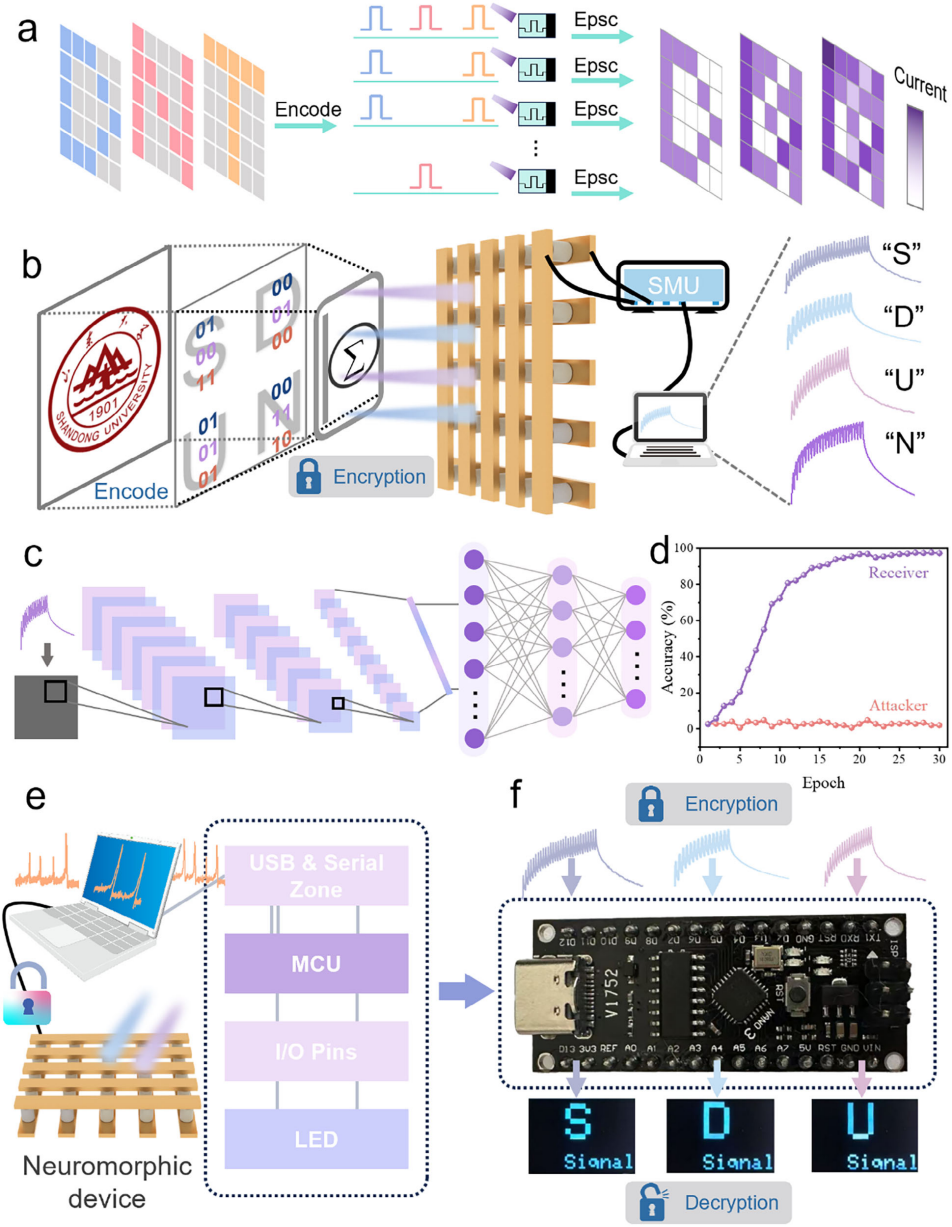
(a) Demonstration of writing multiple letters (“D”, “H”, “T”) into the same device matrix. (b) Multi‐dimensional encryption process for the letters “S”, “D”, “U”, and “N” implemented using the neuromorphic device. (c) Schematic diagram of the constructed convolutional neural network (CNN). (d) Recognition accuracy of the pre‐trained neural network for the obtained electrical signals, with the inset showing a partial confusion matrix at the 30th epoch. (e) Architecture diagram of the implemented mobile secure communication system. (f) The acquired electrical signals are input into a microcontroller pre‐loaded with the neural network's trained weight parameters, and the recognition result is displayed on an externally connected LED screen.

Furthermore, Figure 4a demonstrates the patterned imaging of multiple letters (“D”, “N”, “T”) on a 5 × 5 device array. Utilizing the same encoding and mapping rules described earlier, optical pulses with varying wavelengths, durations, and numbers were applied to the device array in combination with masks. By capturing the current response of each device, a mixed imaging result was generated. This outcome further confirms the device's ability to distinguish optical spike stimuli based on different combinations of physical parameters. To better illustrate the potential of this device‐level, multi‐dimensional encryption scheme for secure communication applications, we attempted to implement it for practical information transmission. As shown in Figure 4b, the entire process of information encoding, conversion, encryption, and acquisition is displayed. Each letter is uniquely mapped to a set of optical pulse sequences based on its 6‐bit ASCII code. These optical pulse sequences are then applied to the device, and the corresponding current responses are collected by a source meter and displayed on a computer. The rightmost section of Figure 4b shows the electrical signals corresponding to the letters “S”, “D”, “U”, and “N”.

Decryption is a critical step in encrypted communication. In this work, a convolutional neural network (CNN) was employed for data processing and recognition. Multiple sets of electrical signals corresponding to all 26 English letters were preprocessed and used as input to train and test the CNN. The architecture of the CNN is shown in Figure 4c. The training results of the neural network are presented in Figure 4d. For trusted information recipients, the convolutional neural network achieved a recognition accuracy of 97.4% after 30 training epochs. The variation in the loss rate and the confusion matrix at the 30th epoch are presented in Figures S17 and S18, respectively. These results demonstrate that legitimate recipients can accurately decrypt the transmitted information. In contrast, for attackers attempting to cracked the encryption, no any improvement in recognition accuracy was observed over the 30 epochs. The average accuracy remained at only 2.88%, equivalent to random guessing probability (1/26), confirming the robustness of the multi‐dimensional encryption scheme. It is worth noting that the use of a neural network for decryption offers significant advantages: as long as the mapping rules (as shown in Figure 4b–d) and the original device are available, the encryption rules can be customized by simply retraining the network with newly collected data. This allows the sender to fully customize the encryption mapping, enhancing both the versatility and security of the scheme. It is also worth noting that since the photocurrent response of the device is solely determined by its internal physical mechanisms once the optical pulse sequence is defined, this multi‐dimensional encryption method theoretically cannot be cracked by mathematical algorithms seeking vulnerabilities.

To improve the portability of the multi‐dimensional encryption application, we attempted to deploy the trained neural network onto a microcontroller. When the microcontroller receives electrical signals from a computer, it can perform recognition and decryption, with the results displayed on an external peripheral device. Figure 4e illustrates the proposed architecture of this system. The complete flowchart of multidimensional encryption is shown in Figure S19, including the processes of sending, encrypting, decrypting, and receiving. As a proof of concept, we used an Arduino Nano V3.0 development board and deployed the trained neural network onto it (considering the computational and memory limitations of the board, only the weight parameters of the neural network were transplanted). The waveforms corresponding to the letters “S”, “D”, and “U” were transmitted to the microcontroller, and the recognition results were successfully displayed on an external LED screen (as shown in Figure 4f). This demonstrates the feasibility of implementing secure communication on mobile devices, significantly enhancing the portability of the application.

**FIGURE 5:**
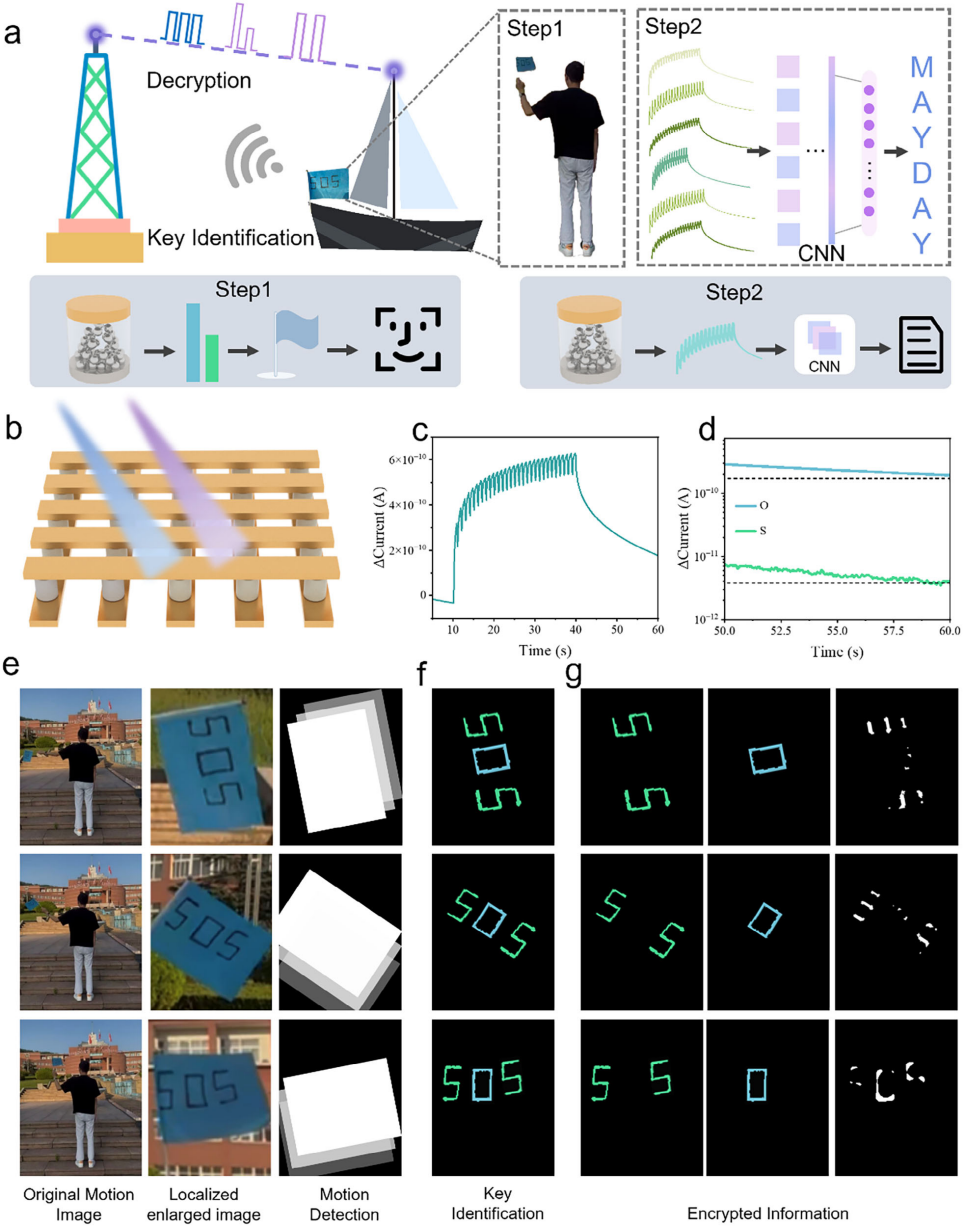
(a) Schematic diagram of encryption scenarios and encryption principles. (b) Device schematic diagram. (c) The mapped current data of the letter “O”. (d) The final state current data of the letters “S” and “O”. (e–g) The key identification process is visualized in phases (The displayed letter is a keyfram/e of dynamic data).

While we have successfully implemented information encryption based on optoelectronic neuromorphic devices and conducted essential proof‐of‐principle validation for their deployment on mobile platforms, their practicality has not yet been fully demonstrated, particularly in complex maritime communication scenarios. Such environments involve extensive information exchange and uncertain receiver identities, making it necessary to construct an additional “friend‐or‐foe identification” communication system on top of the existing framework. Given the conditions of maritime communication, we naturally considered improving upon the commonly used flag semaphore system by integrating it with our neuromorphic devices to serve as a pre‐authentication mechanism. We conceived an application scenario as illustrated in Figure 5a. A complete communication cycle consists of two parts: pre‐authentication for identity verification and multiband encryption for information transmission, both of which are closely associated with neuromorphic devices. The information recognition architecture is shown in Figure S20. First, the person intending to initiate encrypted communication sends signals via semaphore to the receiver. Only after the receiver completes feature recognition can they proceed to identify the actual ciphertext content. This corresponds to the pre‐verification of identity information demonstrated in Step 1, where only a third party who fully understands the semaphore signals can continue with the subsequent multiband encrypted communication. Step 2 illustrates the process based on multiband encryption, where only the encrypted receiver holding the secure physical primitive can decipher the encrypted “MAYDAY”. The principle of multi‐band encryption for generating ciphertext has been explained previously (Figure 5c shows the response waveform of the letter “O”). Here, we focus on explaining the key generation mechanism. Taking the transmission of letters “S” and “O” as examples, we utilized the current responses corresponding to different letters under multi‐band encryption and mapped their current values at the 60‐s mark to the flag signals. Only those who possess the signal‐response mapping rules can qualify as legitimate receivers. Figure 5d displays the steady‐state current values for the letters “S” and “O”.

To clearly demonstrate the key identification mechanism, we present a phased visualization of this process in Figure 5e–g, corresponding to the original motion images, locally amplified postural details, and a schematic diagram of the action recognition system, respectively. Leveraging the efficient temporal signal processing capabilities of reservoir computing, we designed a computational framework specifically for the recognition of dynamic flag semaphore trajectories. The core of this system is a “dynamic reservoir” that nonlinearly maps continuous flag movement trajectories into a high‐dimensional state space, enabling real‐time and accurate recognition of flag semaphore characters. Furthermore, we embedded an information hiding mechanism based on optoelectronic neuromorphic devices into the recognition system. The second column of Figure 5e visualizes the results of encoding device current response information into flag movements. By embedding the steady‐state current data corresponding to different letters into the reservoir computing structure, the system can simultaneously recognize flag movements and verify the hidden information. For example, Figure 5f demonstrates the complete recognition and verification process for the flag signal “SOS” and its corresponding hidden information. It should be pointed out that the reservoir calculation algorithm used for motion flag recognition cannot recognize static “SOS” fonts, and the displayed letters are only a keyframe of dynamic data for illustration.

This design ensures that only communication parties possessing the complete mapping rules and encryption/decryption credentials can achieve effective mutual identification and information recovery. In contrast, for parties lacking the necessary credentials or having only partial credentials, the information extracted by the system appears incomplete or entirely unrecognizable, as shown in Figure 5g, where only partial characters are identified or recognition fails entirely. Thereby, the security and access reliability of communication are effectively ensured.

## 3 Conclusion

In summary, this work successfully develops and demonstrates a hardware‐based multi‐dimensional encryption system centered on an optoelectronic neuromorphic device. The core of this system is a fabricated synaptic device that not only simulates key biological synaptic functions with ultra‐low power consumption but also exhibits distinct, deterministic responses to multi‐parameter optical stimuli. This unique combination of neuro‐mimetic dynamics and multi‐wavelength sensitivity provides the physical foundation for a novel encryption scheme, where information is transformed and secured through the device's intrinsic physical properties rather than purely mathematical algorithms. We have established a complete encryption‐decryption pipeline: information is first encoded into optical pulses defined by wavelength, duration, and number based on ASCII mapping; these pulses are then applied to the device to generate unique, non‐replicable current responses for encryption; finally, a CNN is employed to accurately decrypt the information for authorized recipients. The significant disparity in decryption accuracy between legitimate users (97.40%) and attackers (2.88%) unequivocally validates the high security and robustness of our approach. Beyond theoretical verification, we advanced the system's practicality along two critical paths: First, by implementing the decryption network on a microcontroller, we demonstrated the feasibility of secure, portable communication. Second, and more significantly, we expanded the system's functionality to address dynamic and adversarial environments by constructing a “friend‐or‐foe” identification layer. This pre‐authentication mechanism, which leverages reservoir computing to dynamically decode flag semaphore trajectories and verify hidden information, ensures that only qualified parties can initiate the subsequent encrypted communication cycle. This two‐phase protocol marks a significant step from a standalone encryption device toward a comprehensive and context‐aware secure communication system.

## 4 Experimental Section

### 4.1 Fabrication

The ALD process was used to fabricate the HfAlO_x_‐based RRAM in reaction chamber at temperature of 200°C. One cycle deposition of HfAlO_x_ film consisted of one cycle of HfO_2_ and one cycle of Al_2_O_3_ based on the precursors of TDMAH, TMA and H_2_O. The temperature of TDMAH, TMA and H_2_O sources were kept at 75°C, 25°C, and 25°C, respectively. The N_2_ gas was used as the carrier gas in the reaction. The complete deposition process of one cycle HfAlO_x_ consisted of 1.6 s TDMAH pulse/8 s N_2_ purge/0.2 s H_2_O pulse/8 s N_2_ purge/0.1 s TMA pulse/8 s N_2_ purge/0.2 s H_2_O pulse/8 s N_2_ purge. The NbO_x_ and ITO top electrodes were both prepared using magnetron sputtering.

### 4.2 Device Characterizations

The composition of the device was determined by X‐ray Photoelectron Spectroscopy (XPS). Electrical measurements were carried out using a semiconductor parameter analyzer (Agilent B1500A) under dark conditions. Electrical pulses were produced using a semiconductor pulse generator unit (SPGU) module. Light pulses with tunable wavelength were produced using a xenon lamp system.

## Conflicts of Interest

The authors declare no conflicts of interest.

## Supporting information




**Supporting File**: advs74563‐sup‐0001‐SuppMat.docx.

## Data Availability

The data that support the findings of this study are available from the corresponding author upon reasonable request.
