# Supplementary material for: Low Power Optoelectronic Neuromorphic Memristor for In‐Sensor Computing and Multilevel Hardware Security Communications
Source: Adv Sci (Weinh). 2026 Mar 9;13(26):e24299. doi: 10.1002/advs.202524299 (PMC13159105; doi:10.1002/advs.202524299)
Supplement: Supplementary file 1 — Supporting File: advs74563‐sup‐0001‐SuppMat.docx. [file ADVS-13-e24299-s001.docx]

**Supporting Information**

**Low Power Optoelectronic Neuromorphic Memristor for In-sensor Computing and Multilevel Hardware Security Communications**

Bo Sun^1,2,3^, Jinhao Zhang^1,2,3^, Jialin Meng^1,2,3,4*^, and Tianyu Wang^1,2,3,4,5*^

*^1^Shandong Key Laboratory of Next-Generation Semiconductor Technology and Systems, School of Integrated Circuits, Shandong University, Jinan 250100, China;*

*^2^Shenzhen Research Institute of Shandong University, Shenzhen 518100, China;*

*^3^Suzhou Research Institute of Shandong University, Suzhou, 215123, China;*

*^4^National Integrated Circuit Innovation Center, Shanghai 201203, China;*

*^5^State Key Laboratory of Crystal Materials, Shandong University, Jinan, 250100, China*

**Email: jlmeng@sdu.edu.cn;* [*tywang@sdu.edu.cn*](mailto:tywang@sdu.edu.cn)

**The supporting information file includes:**

Figure S1. Device fabrication process diagram.

Figure S2. The XPS characterization results.

Figure S3. (a) Paired-pulse facilitation (PPF) characteristics of the device. (b) Spike-number-dependent plasticity (SNDP) triggered by a series of ultraviolet (UV) light pulses. (c) Spike-width-dependent plasticity (SWDP). (d) Spike-rate-dependent plasticity (SRDP). (e) Transition of the device from short-term plasticity (STP) to long-term plasticity (LTP).

Figure S4. Paired-pulse test results of the device.

Figure S5. Test results under varying numbers of optical pulses.

Figure S6. Test results under varying optical pulse widths.

Figure S7. Test of the device's transition process from STP to LTP.

Figure S8. Results obtained from direct mapping based on the ASCII code table (A-I).

Figure S9. Results obtained from direct mapping based on the ASCII code table (J-R).

Figure S10. Results obtained from direct mapping based on the ASCII code table (S-Z).

Figure S11. Test results of single pulses with varying durations.

Figure S12. Correspondence and mapping relationships between binary encoding and different physical parameters.

Figure S13. Test results of corresponding letters in multi-dimensional encryption (A-I).

Figure S14. Test results of corresponding letters in multi-dimensional encryption (J-R).

Figure S15. Test results of corresponding letters in multi-dimensional encryption (S-Z).

Figure S16. Visualization results of letters "S" and "D" on the device array.

Figure S17. The variation curve of the loss function during the neural network training process.

Figure S18. The complete confusion matrix of the 30th epoch of the convolutional neural network.

Figure S19. The complete flowchart of multi-dimensional encryption, including the process of sending, encrypting, decrypting, and receiving.

Figure S20. Encryption Architecture Diagram.

Figure S21. “MAYDAY” encryption conversion.

Table 1. Comparison table of energy consumption for different jobs.


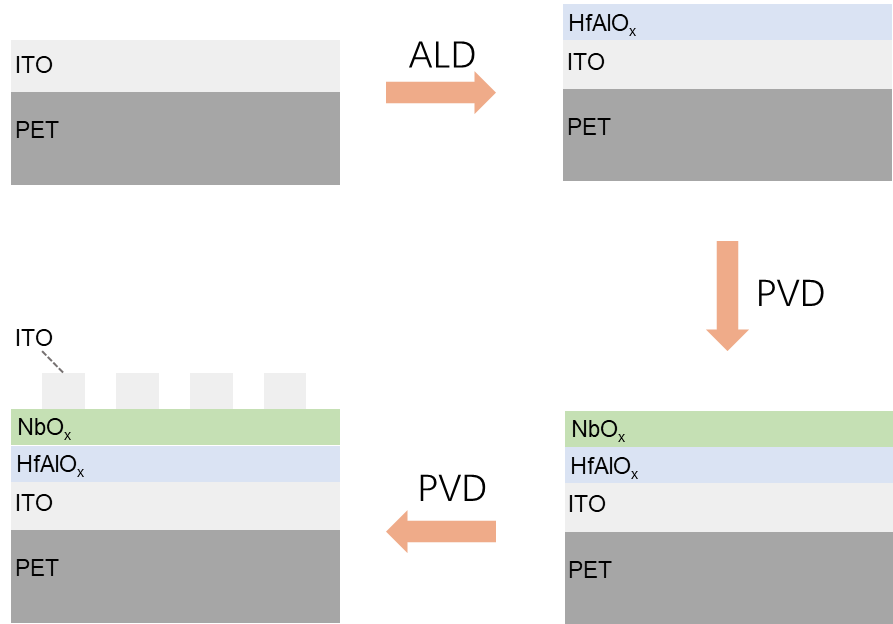


**Figure S1.** Device fabrication process diagram.


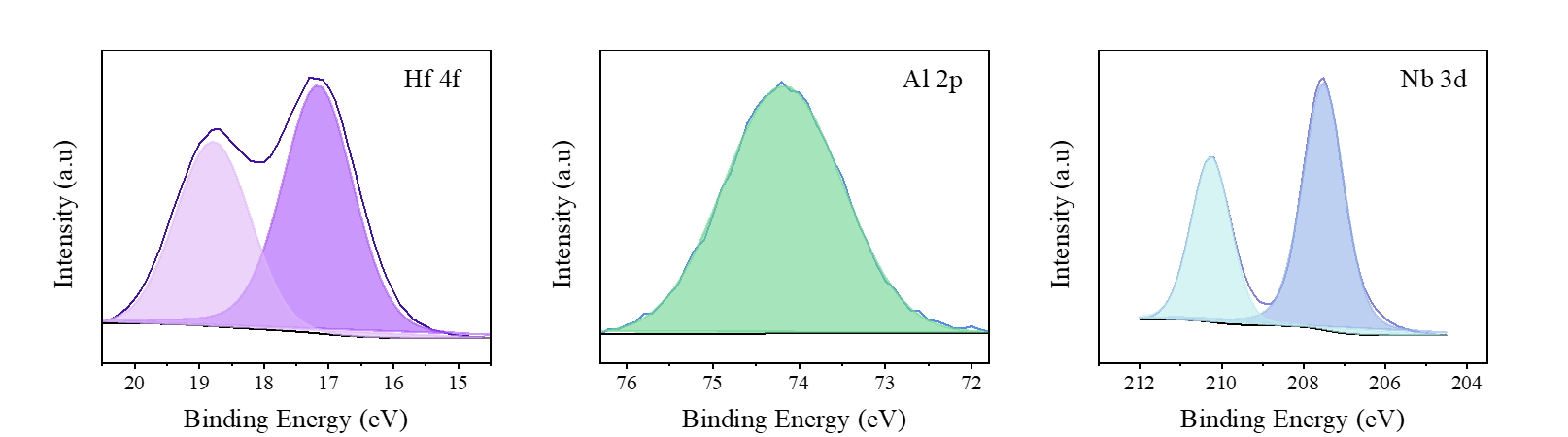


**Figure S2.** The XPS characterization results are presented. The high-resolution spectra reveal the chemical states and elemental composition of the functional layers in the PET/ITO/HfAlOx/NbOx/ITO structure.


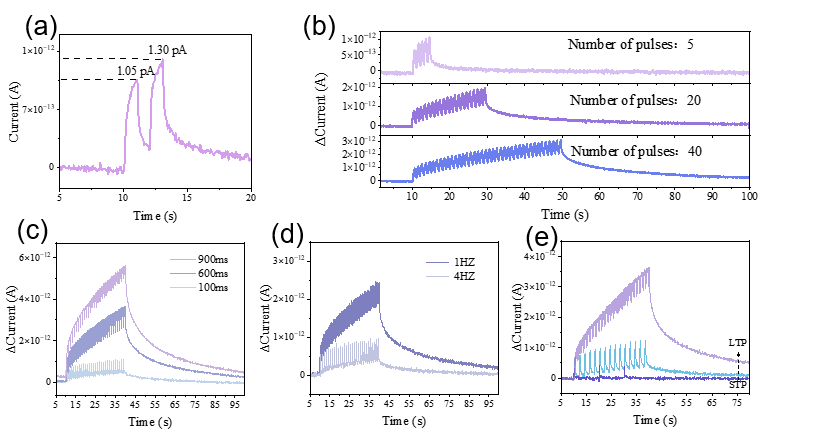


**Figure S3.** (a) Paired-pulse facilitation (PPF) characteristics of the device. (b) Spike-number-dependent plasticity (SNDP) triggered by a series of ultraviolet (UV) light pulses. (c) Spike-width-dependent plasticity (SWDP). (d) Spike-rate-dependent plasticity (SRDP). (e) Transition of the device from short-term plasticity (STP) to long-term plasticity (LTP).


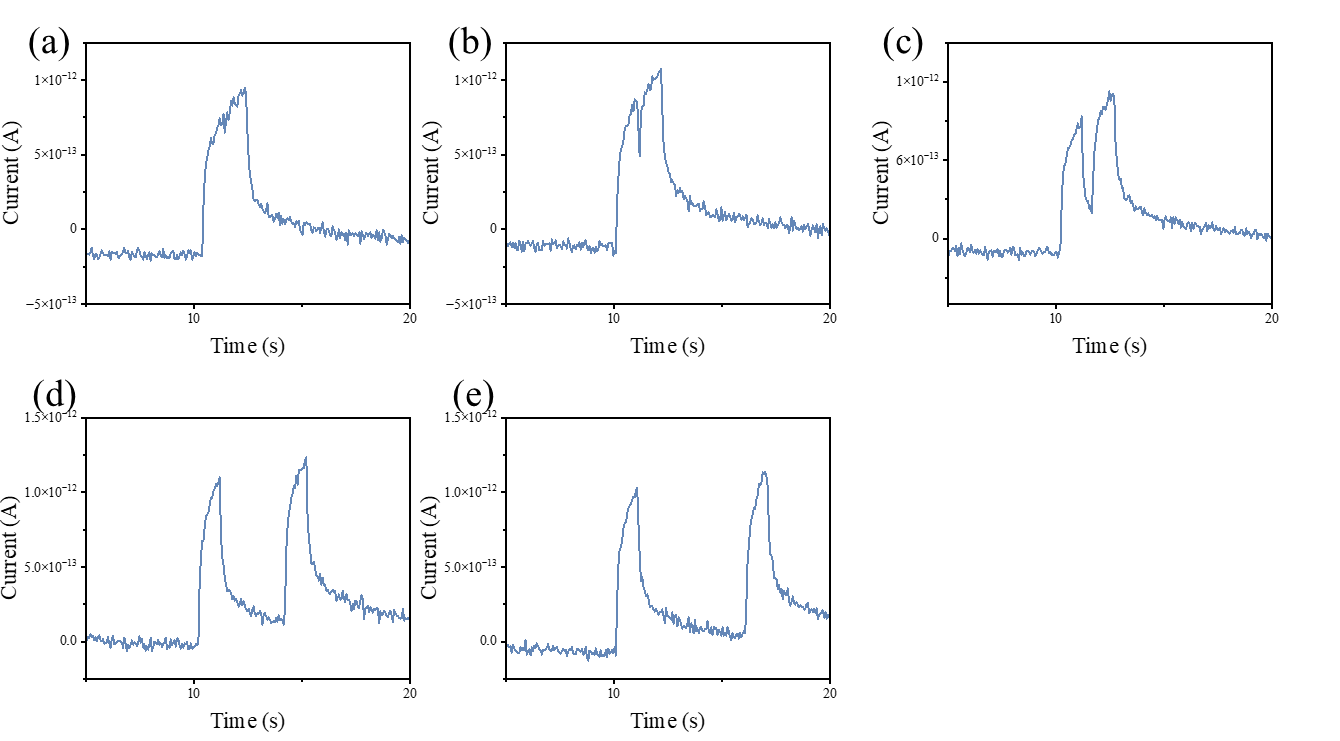


**Figure S4.** Paired-pulse test results of the device.


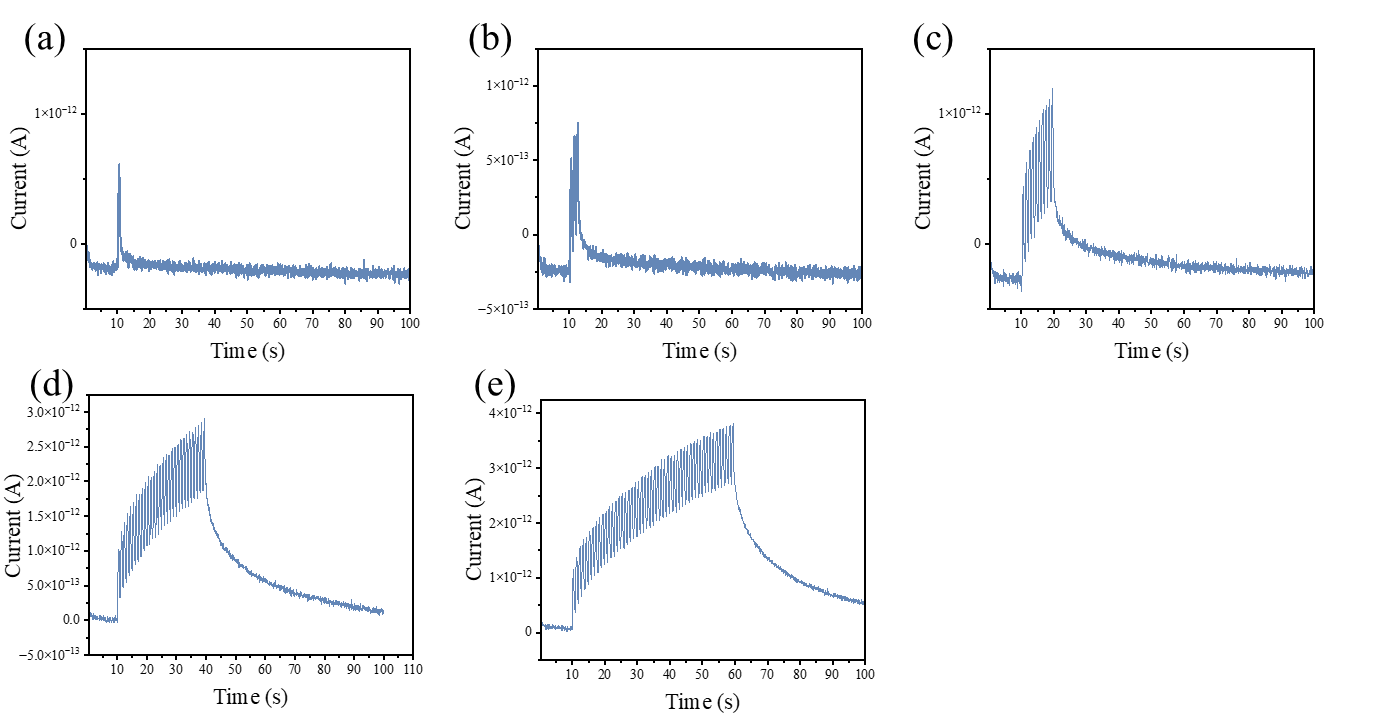


**Figure S5.** Test results under varying numbers of optical pulses.


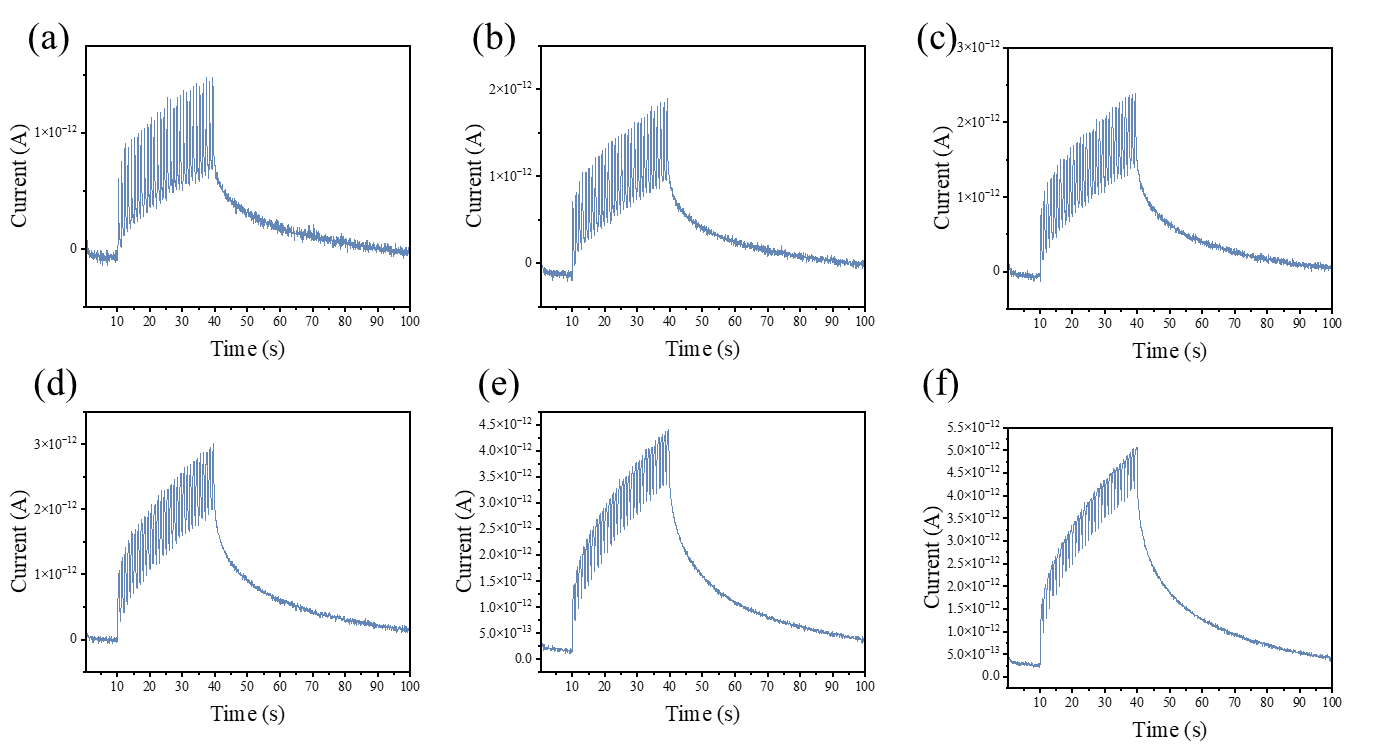


**Figure S6.** Test results under varying optical pulse widths.


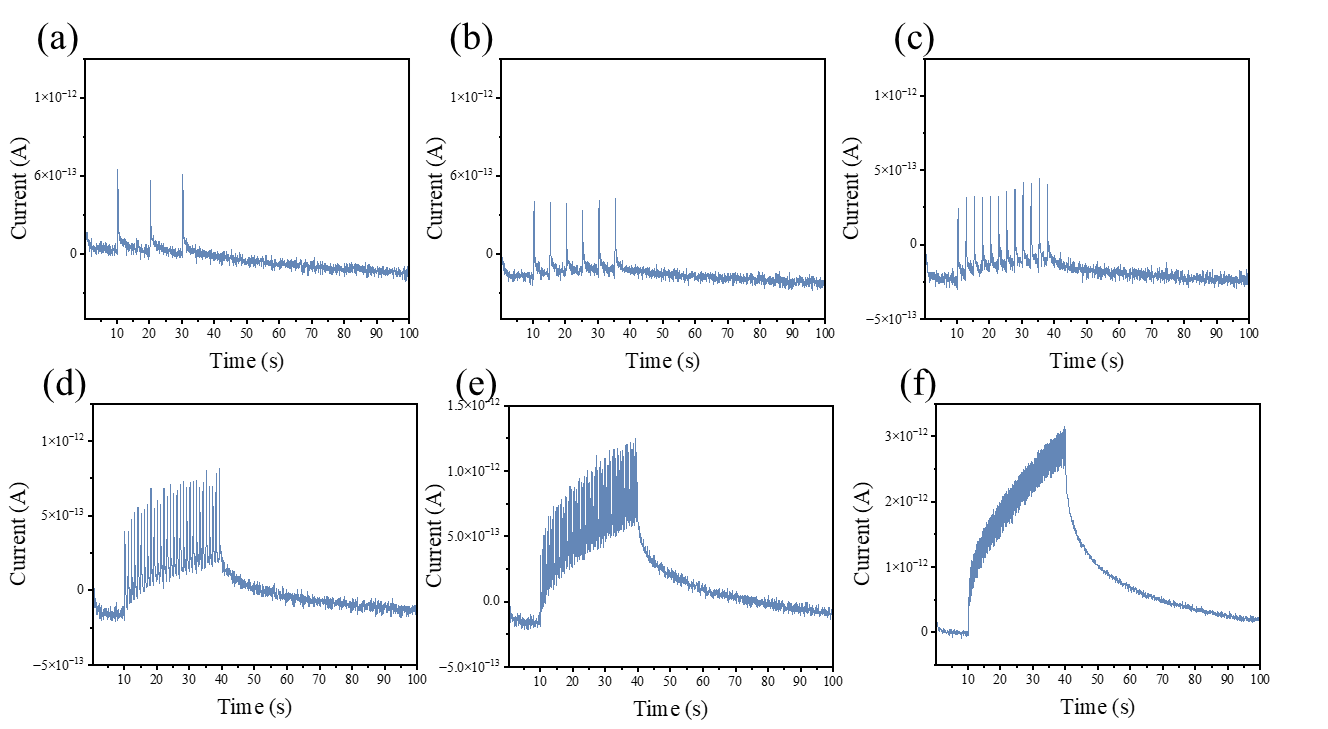


**Figure S7.** Test of the device's transition process from STP to LTP.


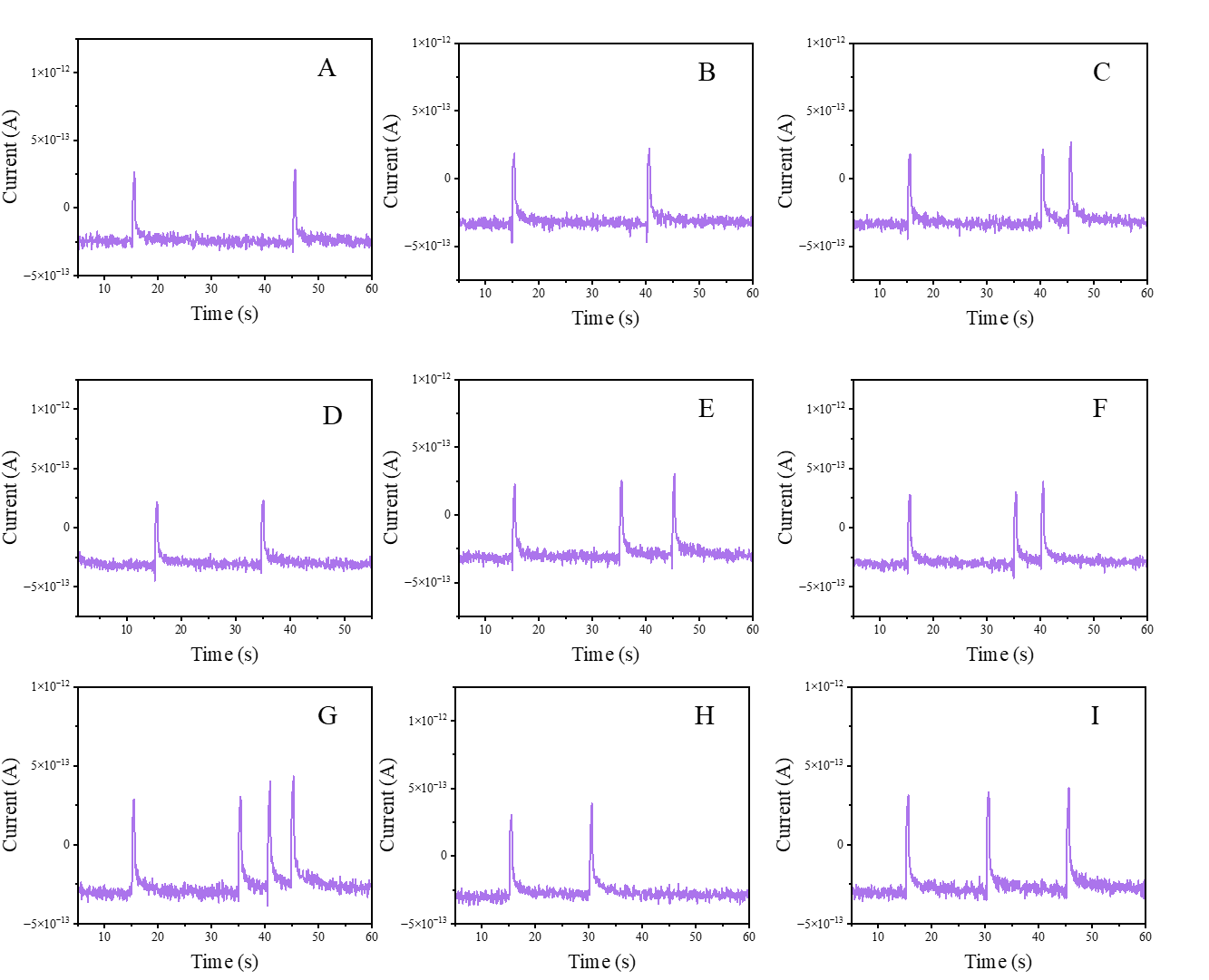


**Figure S8.** Results obtained from direct mapping based on the ASCII code table (A-I).


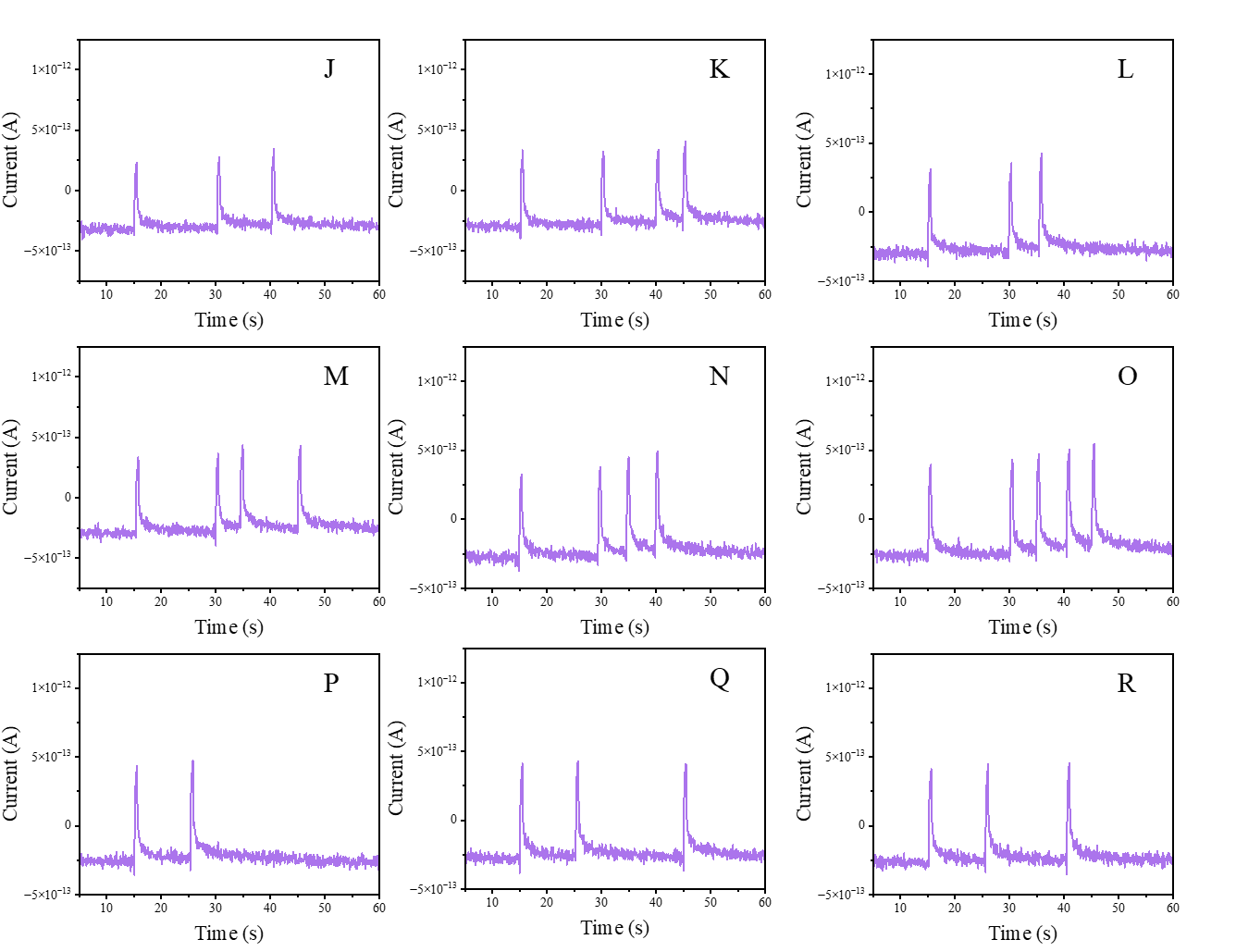


**Figure S9.** Results obtained from direct mapping based on the ASCII code table (J-R).


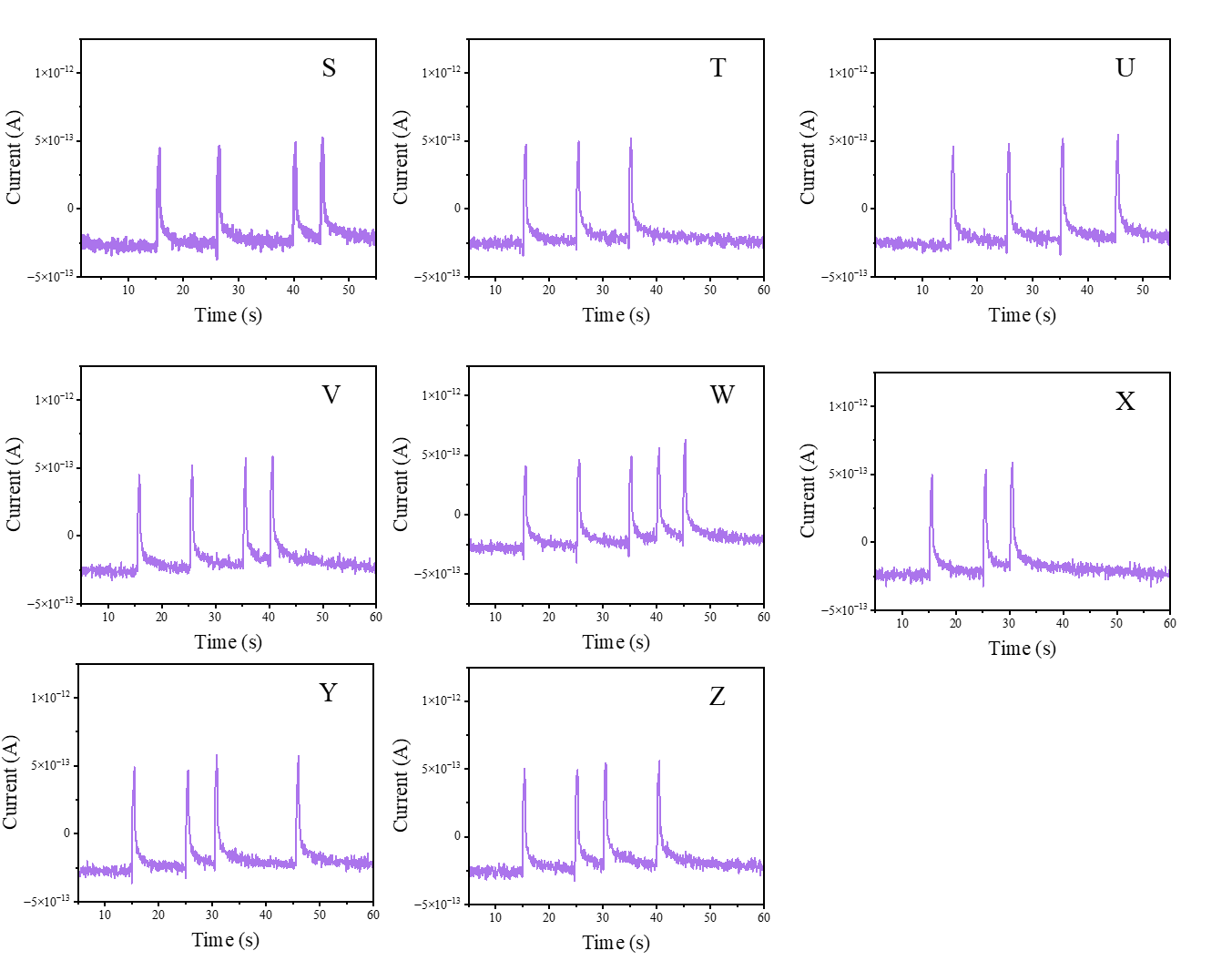


**Figure S10.** Results obtained from direct mapping based on the ASCII code table (S-Z).


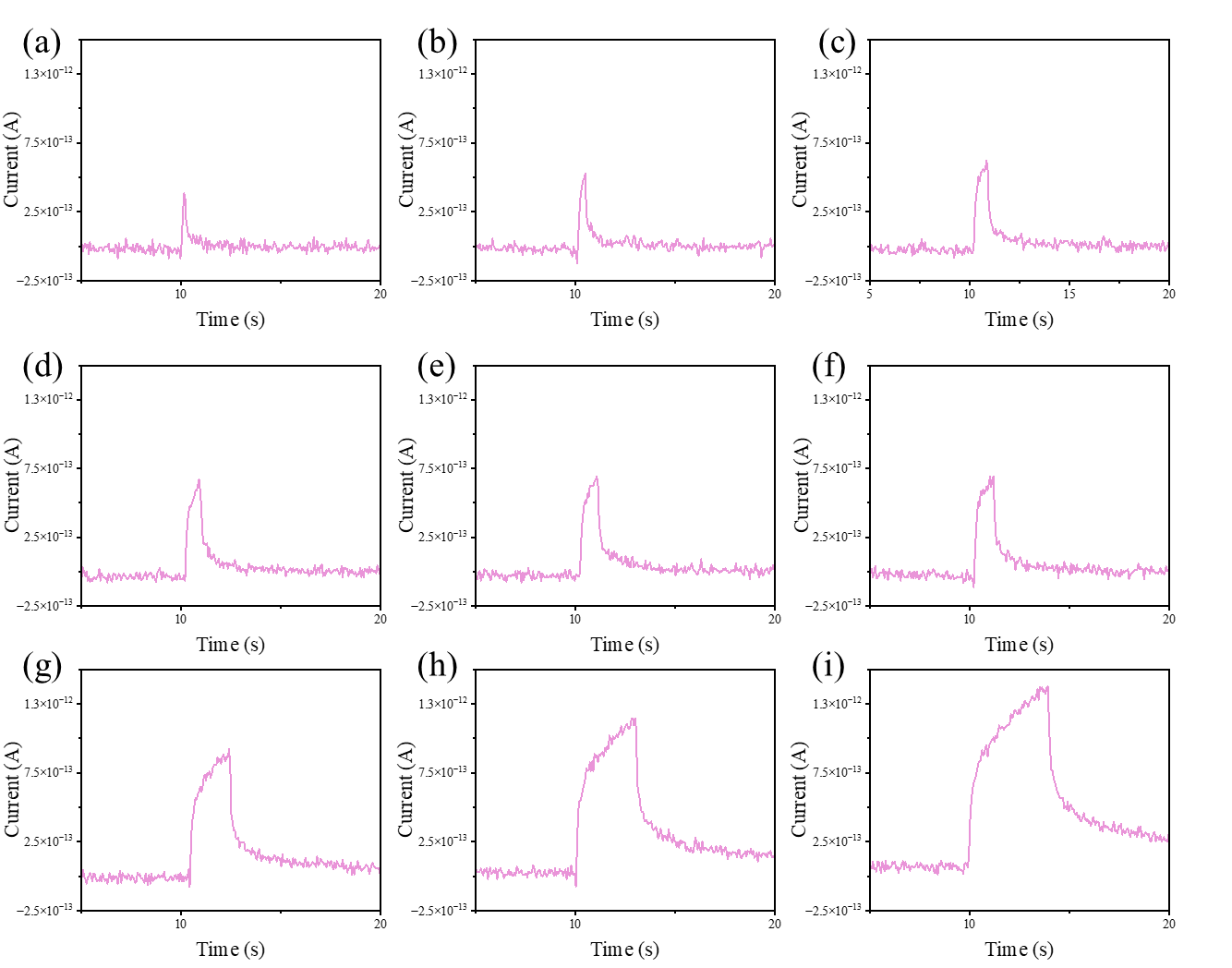


**Figure S11.** Test results of single pulses with varying durations.


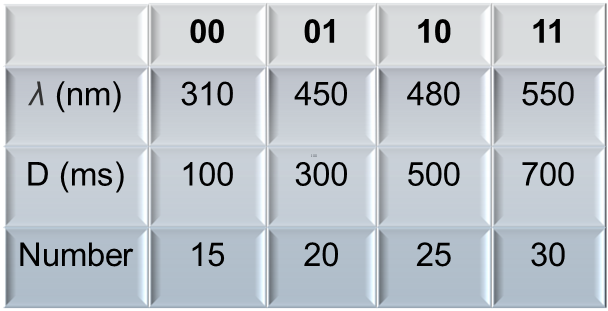


**Figure S12.** Correspondence and mapping relationships between binary encoding and different physical parameters.


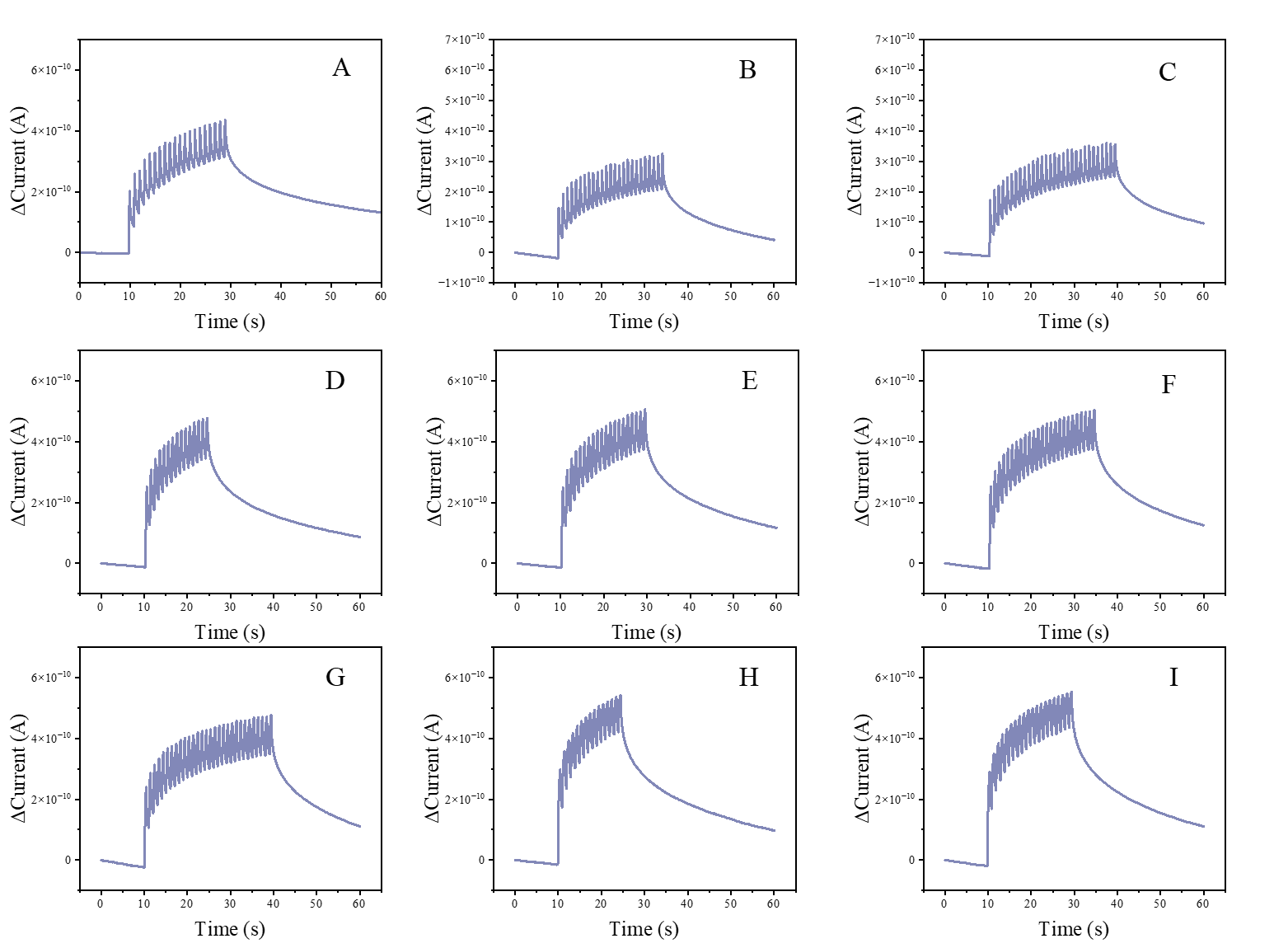


**Figure S13.** Test results of corresponding letters in multi-dimensional encryption (A-I).


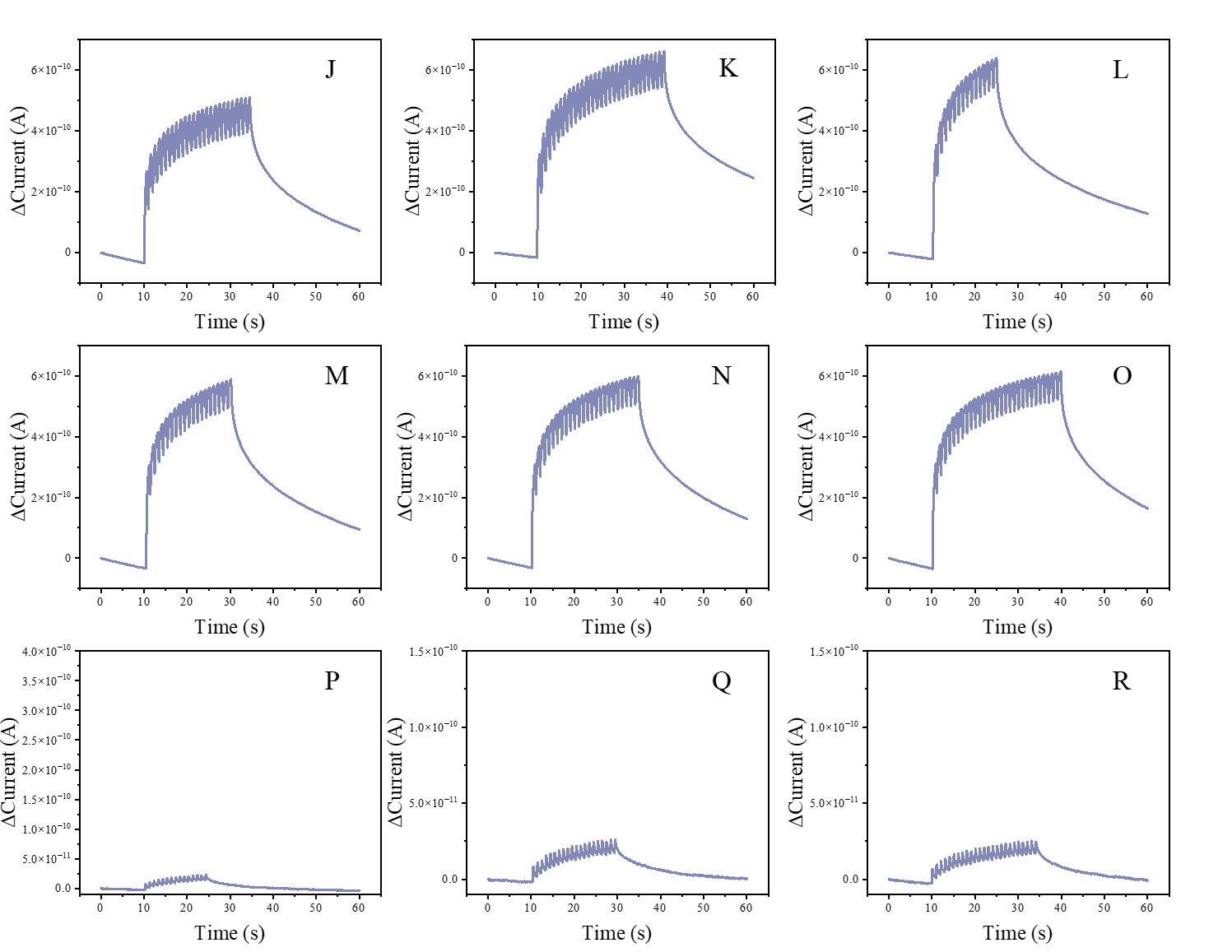


**Figure S14.** Test results of corresponding letters in multi-dimensional encryption (J-R).


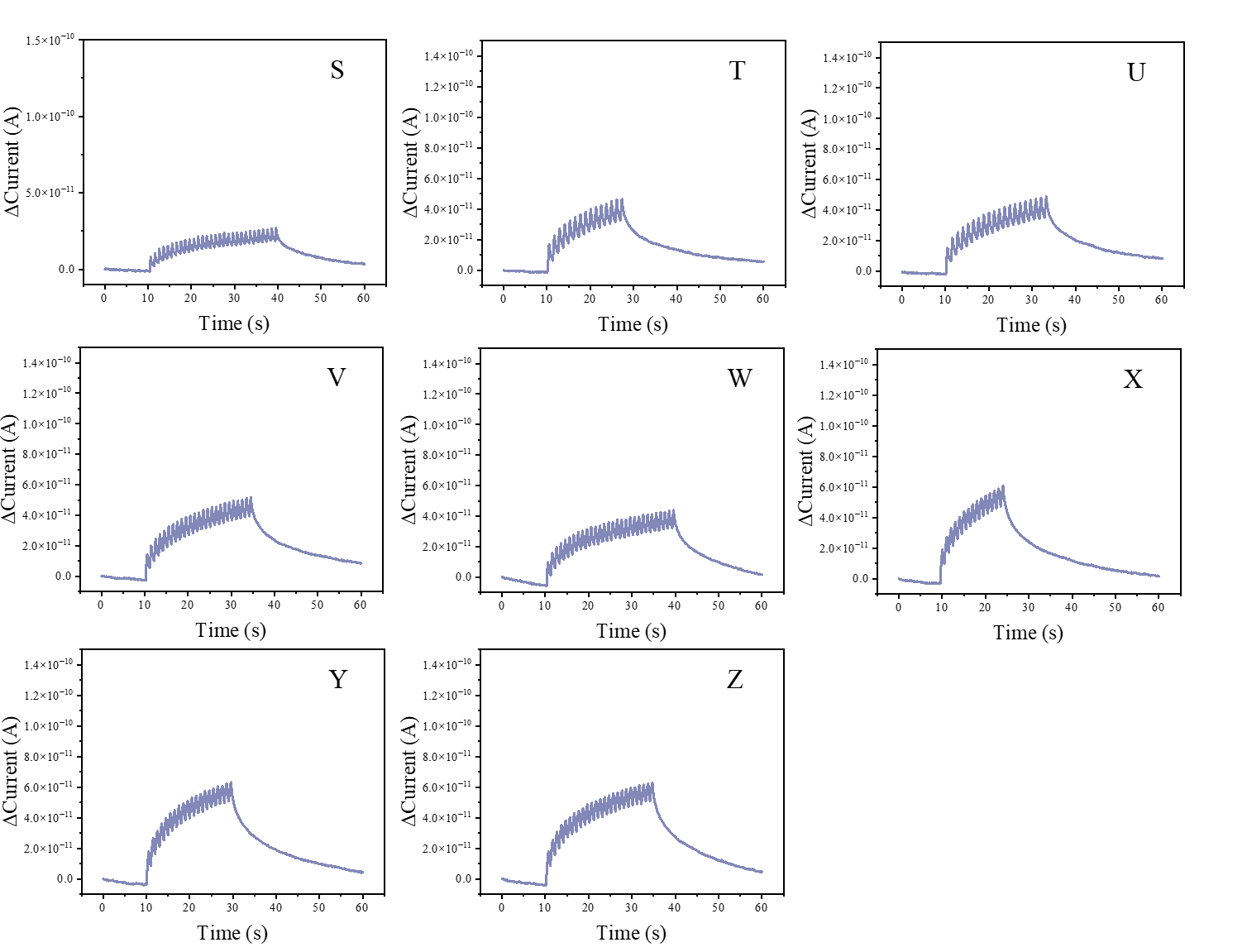


**Figure S15.** Test results of corresponding letters in multi-dimensional encryption (S-Z).


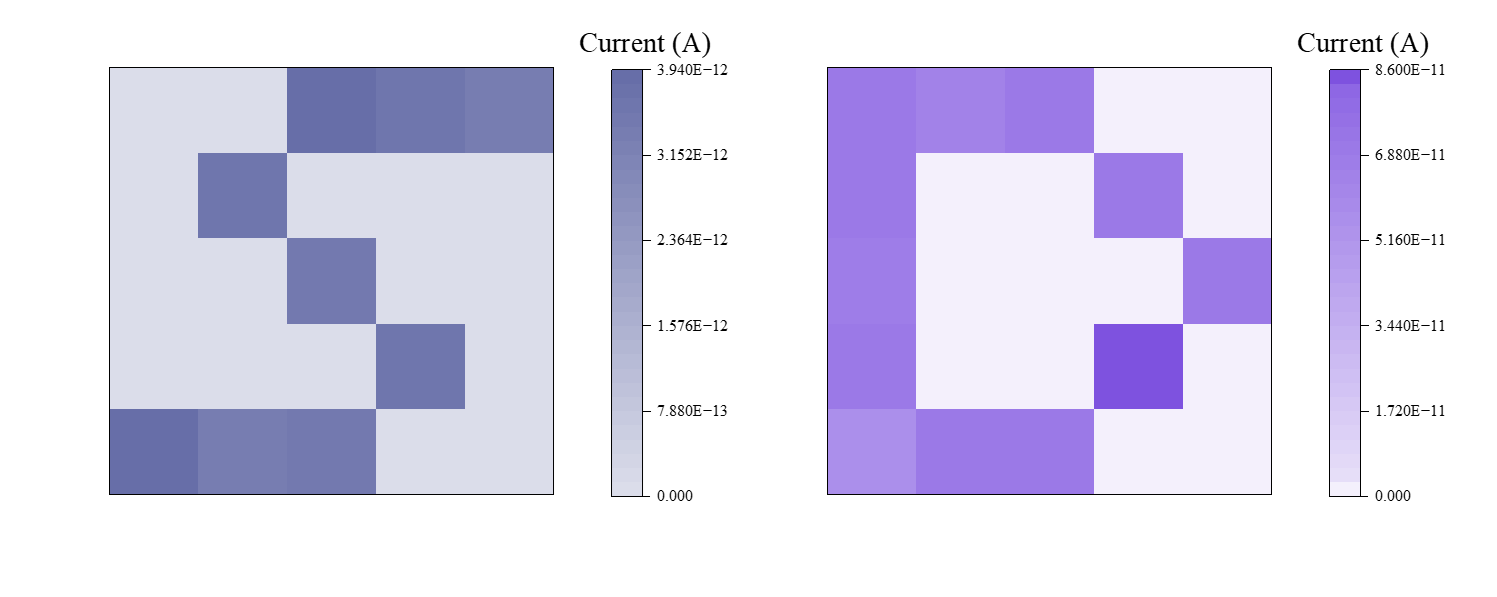


**Figure S16.** Visualization results of letters "S" and "D" on the device array.

**Figure S17.** The variation curve of the loss function during the neural network training process.

**Figure S18.** The complete confusion matrix of the 30th epoch of the convolutional neural network. This result comprehensively reflects the recognition performance of the neural network for all 26 letters (A-Z) after training.


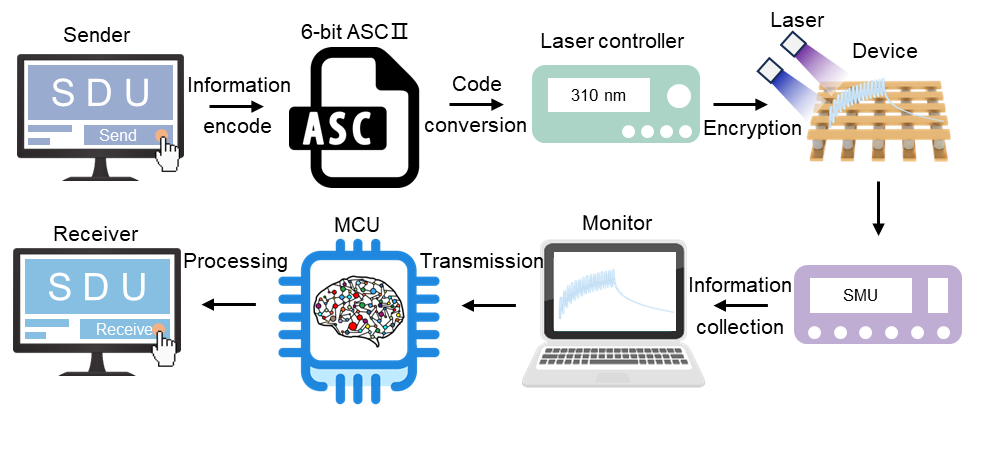


**Figure S19.** The complete flowchart of multi-dimensional encryption, including the process of sending, encrypting, decrypting, and receiving.


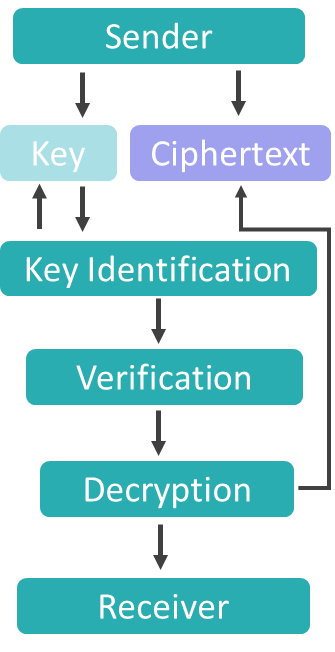


**Figure S20.** Encryption Architecture Diagram.


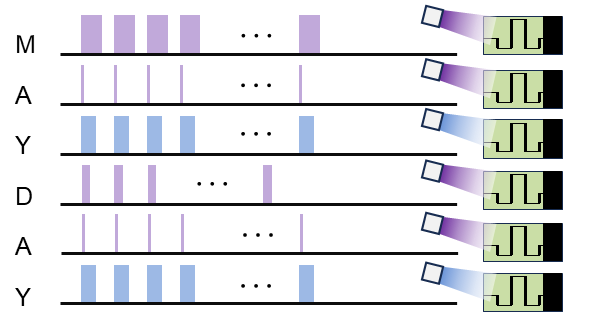


**Figure S21.** “MAYDAY” encryption conversion.

| Structure | *E/Spike* | Years | Reference |
| --- | --- | --- | --- |
| Al/InAlZnO nanofibers/Al | 75 fj | 2024 | [1] |
| graphene/(Al,Ga)N nanowire heterojunction | 25 fj | 2025 | [2] |
| MoS_2_/Nb_2_O_5_  heterostructure | 6 pj | 2021 | [3] |
| ReS₂/h-BN/Gra vdW heterostructure | 500 fj | 2025 | [4] |
| perovskite  nanowire | 500fj | 2024 | [5] |
| BP/CdS heterostructure | 4.78 fj | 2022 | [6] |
| HfAlO_x_/NbO_x_ | 3.3 fj | 2025 | Our |

**Table 1**. Comparison table of energy consumption for different jobs.

Reference

[1] W. Xiao, Y. Dong, R. Ci, G. Liu, F. Shan, "Oxygen Vacancy-Induced Synaptic Plasticity in InAlZnO Nanofiber Transistors for Low-Power Neuromorphic Electronics," *IEEE Trans. Electron Devices* **2024**, *71* (10), 6430, <https://doi.org/10.1109/ted.2024.3449828>.

[2] M. Jiang, Y. Zhao, T. Liu, Y. Chang, Y. Tang, M. Zhou, Y. Shi, J. Zhang, L. Bian, S. Lu, "A dual-mode transparent device for 360° quasi-omnidirectional self-driven photodetection and efficient ultralow-power neuromorphic computing," *Light Sci. Appl.* **2025**, *14* (1), <https://doi.org/10.1038/s41377-025-01991-y>.

[3] J. H. Nam, S. Oh, H. Y. Jang, O. Kwon, H. Park, W. Park, J. D. Kwon, Y. Kim, B. Cho, "Low Power MoS2/Nb2O5 Memtransistor Device with Highly Reliable Heterosynaptic Plasticity," *Adv. Funct. Mater.* **2021**, *31* (40), <https://doi.org/10.1002/adfm.202104174>.

[4] Z. Yang, S. Huo, Z. Zhang, F. Meng, B. Liu, Y. Wang, Y. Ma, Z. Wang, J. Xu, Q. Tian, Y. Wang, Y. Ding, X. Hu, Y. Xie, S. Fan, C. Pan, E. Wu, "High‐Precision Multibit Opto‐Electronic Synapses Based on ReS2/h‐BN/Graphene Heterostructure for Energy‐Efficient and High‐Accuracy Neuromorphic Computing," *Adv. Funct. Mater.* **2025**, *35* (48), <https://doi.org/10.1002/adfm.202509119>.

[5] Q. Liu, P. Wang, Q. Wei, L. Zhou, H. Ren, C. Wang, J. Peng, L. Zhao, M. Li, "Chiral Perovskite Nanowire Optoelectronic Synapse for Full‐Stokes Polarization‐Resolved Perception and Reservoir Computing," *Adv. Funct. Mater.* **2024**, *35* (8), <https://doi.org/10.1002/adfm.202415551>.

[6] C. Zhu, H. Liu, W. Wang, L. Xiang, J. Jiang, Q. Shuai, X. Yang, T. Zhang, B. Zheng, H. Wang, D. Li, A. Pan, "Optical synaptic devices with ultra-low power consumption for neuromorphic computing," *Light Sci. Appl.* **2022**, *11* (1), <https://doi.org/10.1038/s41377-022-01031-z>.
